# Renoprotective capacities of non-erythropoietic EPO derivative, ARA290, following renal ischemia/reperfusion injury

**DOI:** 10.1186/1479-5876-11-286

**Published:** 2013-11-13

**Authors:** Willem G van Rijt, Gertrude J Nieuwenhuijs-Moeke, Harry van Goor, Petra J Ottens, Rutger J Ploeg, Henri GD Leuvenink

**Affiliations:** 1 Department of Surgery, University Medical Center Groningen, Hanzeplein 1, 9713, GZ Groningen, The Netherlands; 2 Department of Pathology and Medical Biology, University Medical Center Groningen, Groningen, The Netherlands; 3 Department of Anaesthesiology, University Medical Center Groningen, University of Groningen, Groningen, The Netherlands; 4 Nuffield Department of Surgical Sciences, University of Oxford, Oxford, UK

**Keywords:** Erythropoietin, Renoprotection, ARA290, Pyroglutamate helix B-surface peptide, Ischemia/reperfusion injury, Renal transplantation

## Abstract

**Background:**

ARA290 is a non-erythropoietic EPO derivative which only binds to the cytoprotective receptor complex (EPOR_2_-βcR_2_) consisting of two EPO-receptors (EPOR) and two β common receptors (βcR). ARA290 is renoprotective in renal ischemia/reperfusion (I/R). In a renal I/R model we focussed on timing of post-reperfusional administration of ARA290. Furthermore, we investigated the anti-inflammatory properties of ARA290.

**Methods:**

Twenty-six male Lewis/HanHsd rats were exposed to unilateral ischemia for 30 minutes, with subsequent removal of the contralateral kidney. Post-reperfusion, ARA290 was administered early (one hour), late (four hours) or repetitive (one and four hours). Saline was used as vehicle treatment. Rats were sacrificed after three days.

**Results:**

Early ARA290 treatment improved renal function. Late- or repetitive treatment tended to improve clinical markers. Furthermore, early ARA290 treatment reduced renal inflammation and acute kidney injury at three days post-reperfusion. Late- or repetitive treatment did not affect inflammation or acute kidney injury.

**Conclusions:**

ARA290 attenuated renal ischemia/reperfusion injury. This study showed the anti-inflammatory effect of ARA290 and suggests early administration in the post-reperfusional phase is most effective. ARA290 is a candidate drug for protection against ischemic injury following renal transplantation.

## Introduction

As a consequence of the relative shortage of donor organs, marginal donor kidneys, for example kidneys donated after circulatory death (DCD), are increasingly used. DCD kidneys are exposed to a variable and extended primary warm ischemic period compared to donor kidneys of brain dead- or living donors. Therefore renal ischemia/reperfusion (I/R) injury is an important cause of short-term dysfunction in DCD kidneys. Renal I/R injury results in an increased incidence of delayed graft function (DGF) of 72% in DCD kidneys versus 18% in kidneys donated after brain death (DBD). In primary non function (PNF) the differences are even more pronounced, respectively 23% versus 4%. Subsequently, the increased incidence of PNF causes reduced graft survival of DCD kidneys [1]. In DCD donation, the occurrence of primary warm ischemia is inevitable; however a substantial part of the damage occurs during the reperfusion phase [2]. This makes cytoprotective treatment early after transplantation an attractive opportunity to improve short- and long-term outcome of kidney transplants.

In pre-clinical studies it has been shown that erythropoietin (EPO), administered post-reperfusion, is able to attenuate renal I/R injury [3]. Therefore, EPO mediated cytoprotection may improve short- and even long-term renal function following kidney transplantation with marginal donor kidneys.

EPO regulates erythropoiesis by binding to the classical, homodimeric complex of two EPO receptors (EPOR_2_) on erythroid progenitor cells, but cytoprotection is mediated by binding of EPO to a heteromeric receptor complex consisting of two EPOR and two β common receptors (EPOR_2_-βcR_2_) [4]. Activation of this protective receptor complex increases janus kinase-2 (JAK-2) phosphorylation, which results in a cascade of anti-inflammatory, anti-apoptotic and pro-survival effects [5,6]. Furthermore, EPO also directly affects renal function. EPO enhances endothelial nitric oxide synthase (eNOS) activity [7,8] which increases vasodilatation of the afferent arterioles. This results in increased glomerular filtration rate [9,10].

However, a major drawback in the use of EPO as a cytoprotective agent is its stimulative effect on erythropoiesis and also thrombopoiesis. An increased serum EPO raises the haematocrit and markedly enhances platelet and endothelial activation [11] is associated with adverse effects, such as thrombotic events which can be life threatening. The binding affinity of the protective EPOR_2_-βcR_2_ complex for EPO is considerably lower compared to the affinity of the classical, erythropoietic EPO receptor complex [12]. Therefore the required dose of EPO for cytoprotection will be relatively high, which increases the risk of cardiovascular adverse events. Based on pre-clinical studies cytoprotective, high dose EPO treatment has been evaluated in four clinical trials. None of these trials were able to show reduced PNF, reduced DGF or improved short-term renal function. One study even observed an increased risk of thrombosis [13-16].

To avoid these adverse events and provide the opportunity to safely administer relatively high doses, EPO derivatives have been developed that only activate the EPOR_2_-βcR_2_ complex and do not stimulate erythropoiesis [17]. ARA290 is a small synthetic peptide, which selectively binds to the EPOR_2_-βcR_2_ complex. It has already been shown that ARA290, also known as pHBSP, has no erythropoietic properties and is renoprotective in a rodent models of renal I/R [17,18]. Mechanistically, ARA290 activates survival pathway AKT and inhibits pro-inflammatory pathway glycogen synthase kinase-3β (GSK-3β) [18]. Recently, we showed the renal protective capacities of ARA290 in a renal I/R model in pigs. ARA290, administered repetitively at zero, two, four and six hours post-reperfusion, improved renal function and reduced structural damage [19].

We hypothesized that post-reperfusional administration of ARA290 reduces inflammation and improves renal function following renal I/R in rats. Three different times of post-reperfusional administration have been tested to investigate the effect of timing of ARA290 treatment. Kidney function, inflammation and renal morphology were studied at three days post-reperfusion.

## Materials and methods

### Animals

Twenty-six male Lewis/HanHsd rats (Harlan, Horst, the Netherlands, 250–300 gr.) were used. They were housed individually with free access to water and rat chow. One rat was excluded because of unsuccessful clamping of the vena and arteria renalis (control group). Two rats died during surgery (control group and repetitive treatment) and one rat has been terminated at day one because of respiratory failure (control group). The animal experiments were approved by the animal ethics committee of the university Groningen (DEC-RuG, 4762B, Groningen, the Netherlands). The experiments were performed according to international and Dutch guidelines of animal research.

### ARA290

ARA290 (ARAIM Pharmaceuticals, Ossining, USA) is a small synthetic peptide consisting of eleven amino acids. It has been derived from the binding site of EPO to the protective EPOR_2_-βcR_2_ complex and it does not bind to the classical EPOR2 complex. The plasma half-life is approximately two minutes.

### Study design

The animals were randomized into four groups and treated at one, four or one and four hours post-reperfusion (Table 1). The used concentration ARA290 was 10 nmol/kg (10 nmol/kg = 12.58 ug/kg) and saline (0.9%) served as vehicle treatment. The control group was vehicle treated at one and four hours post-reperfusion. Times of administration and dosage of ARA290 were chosen based on earlier renal I/R experiments [17,19]. Both ARA290 and saline were injected intraperitoneally. A standardized I/R model was used to determine the effectiveness of ARA290. The warm ischemic time was 30 minutes and the rats were sacrificed 72 hours post-reperfusion.

**Table 1:** Study design

**Group**	**One hr. post-reperfusion**	**Four hrs. post-reperfusion**	**Number of animals**
**Controls**	Saline	Saline	4
**Early treatment**	10 nmol/kg ARA290	Saline	6
**Late treatment**	Saline	10 nmol/kg ARA290	6
**Repetitive treatment**	10 nmol/kg ARA290	10 nmol/kg ARA290	6

### Surgical procedure

All animals were sedated using ketamine (0.75 μl/g) and medetomedine (0.5 μl/g). As an analgesic, buprenorfine was used (pre-operative: 0.005 μl/g, directly post-operative: 0.02 μl/g, 24 hours post-operative: 0.025 μl/g). Once the abdomen was opened, unilateral renal ischemia of 30 minutes was performed by clamping the vena and arteria renalis with non-traumatic clamps. The contralateral kidney was removed during the ischemic period. After 72 hours blood samples were collected under anaesthesia. After this the animals were sacrificed by performing a cardiotomy. Prior to removal of the kidney, it was flushed via the aorta with 10 ml 0.9% NaCl at 4°C.

### Samples

Blood samples were stored at −80°C and serum parameters were measured using standard protocols. A section of the kidney was snap frozen in N_2_ and stored in −80°C. For immunohistochemistry and morphology another section was fixed in 4% formalin and subsequently embedded in paraffin.

### Reverse transcription polymerase chain reaction (qRT-PCR)

RNA was extracted from snap frozen sections of total kidney tissue using Trizol reagent according to the manufacturer’s instructions (Invitrogen, Breda, the Netherlands). Total RNA was treated with DNAse I to remove genomic DNA contamination (Invitrogen, Breda, the Netherlands). The integrity of total RNA was analysed by gel electrophoresis. cDNA synthesis was performed from 1-μg total RNA using M-MLV (Moloney murine leukaemia virus) Reverse Transcriptase and oligo-dT primers (Invitrogen, Breda, The Netherlands).

Primer sets (Table 2) were designed using Primer Express 2.0 software (Applied Biosystems, Foster City, CA). Amplification and detection were performed with the ABI Prism 7900-HT Sequence Detection System (Applied Biosystems) using emission from SYBR green master mix (Applied Biosystems). The PCR reactions were performed in triplicate. After an initial activation step at 50°C for 2 min and a hot start at 95°C for 10 min, PCR cycles consisted of 40 cycles at 95°C for 15 sec and 60°C for 60 sec. Dissociation curve analysis were performed for each reaction to ensure amplification of specific products.

**Table 2:** qRT-PCR primers

**Primer**	**Forward**	**Reverse**	**Amplicon length (bp)**
TNF-α	5′-AGGCTGTCGCTACATCACTGAA-3′	5′-TGACCCGTAGGGCGATTACA-3′	67
IL-6	5′-CCAACTTCCAATGCTCTCCTAATG-3′	5′-TTCAAGTGCTTTCAAGAGTTGGAT-3′	89
Kim-1	5′-AGAGAGAGCAGGACACAGGCTTT-3′	5′-ACCCGTGGTAGTCCCAAACA-3′	75
α-SMA	5′-GAGAAAATGACCCAGATTATGTTTGA-3′	5′-GGACAGCACAGCCTGAATAGC-3′	74

Gene expression of tumour necrosis factor–α (TNF-α), interleukin-6 (IL-6), kidney injury molecule-1 (Kim-1), α-smooth muscle actin (α-SMA) and β-actin (housekeeping gene) were determined. Gene expression was normalized with the mean of b-actin mRNA content. Results were finally expressed as 2^–ΔCT^ (CT = threshold cycle), which is an index of the relative amount of mRNA expression in each tissue.

### Immunohistochemistry

Kidney samples were cut into 4-μm-thick sections. The morphology was evaluated by periodic acid-Schiff (PAS) staining. Immunohistochemical stainings for Kim-1 (acute tubular damage) and α-SMA (pre-fibrotic changes) were performed on paraffinized tissue. HIS-48 (anti-granulocyte antibody) was stained on cryosections.

Deparaffinised sections were subjected to antigen retrieval. For the Kim-1 staining sections were incubated for one night in 0.1 M Tris/HCl buffer (pH 9.0). Antigen retrieval was not necessary for the α-SMA or HIS-48 staining.

Endogeneous peroxidase was blocked by 0.3% H_2_O_2_ for 30 minutes. Kim-1 (1:400, Bonventre), α-SMA (1:400, Abcam, 1A4, Cambridge, UK) and HIS-48 (1:2, Department of pathology and Microbiology, University Medical Center Groningen) antibodies were used. The incubation time of the primary antibodies was 1 hour. For the Kim-1 staining a secondary peroxidase-conjungated goat-anti-rabbit antibody (1:100, DAKO, Glostrup, Denmark) and a tertiary peroxidase-conjungated rabbit-anti-goat antibody (1:100, DAKO, Glostrup, Denmark) were used. For the α-SMA staining we only used a secondary peroxidase-conjungated goat-anti-mouse antibody (1:50, DAKO, Glostrup, Denmark). For the HIS-48 staining a secondary peroxidase-conjungated rabbit-anti-mouse antibody (1:100, DAKO, Glostrup, Denmark) and a tertiary peroxidase-conjungated goat-anti-rabbit antibody (1:100, DAKO, Glostrup, Denmark) were used. Normal rat serum (1:100) was added to the secondary and tertiary antibodies and the incubation time was 30 minutes. Then the peroxidase activity was visualized by ten minutes incubation in 3.3-diaminobenzidine tetrachloride or aminoethylcarbazole for respectively paraffinized- or crysections. Subsequently the sections were counterstained with haematoxylin.

Finally, the sections were scanned using APERIO scanscope (Aperio, Vista, United States). The expression of the immunohistochemical staining of each section was quantified using APERIO image scope software.

### Statistical analyses

All data are presented as mean ± standard error of the mean (SEM). Kruskal-Wallis H test with Dunn’s multiple comparison test as post hoc analyses was used to analyse the data. All groups were compared to the controls. A p < 0.05 was considered significant.

## Results

### Clinical parameters

**Figure 1:**
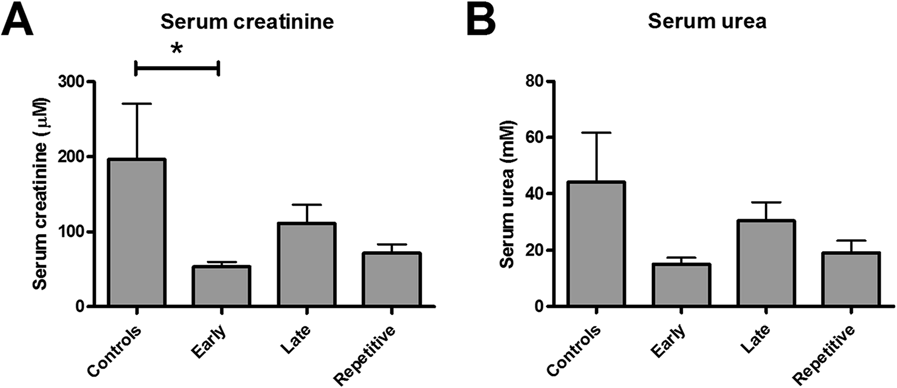
**The effect of ARA290 on markers of renal function.** Early ARA290 treatment significantly reduced serum creatinine levels at three days post-reperfusion. Late- and repetitive ARA290 treatment tended to reduce serum creatinine levels to a lesser extent (**A**, p < 0.05). The treatment effect of ARA290 on serum urea levels showed the same tendency as the effect on serum creatinine levels **(B)**.

Early ARA290 treatment at one hour post-reperfusion significantly reduced serum creatinine levels at three days post-reperfusion compared to the control group. Late or repetitive treatment did not significantly change serum creatinine levels (Figure 1A). Serum urea levels showed a similar tendency although these differences were not significant (Figure 1B). No differences in serum aspartate transaminase (ASAT) or lactate dehydrogenase (LDH) levels were found.

### Inflammation

**Figure 2:**
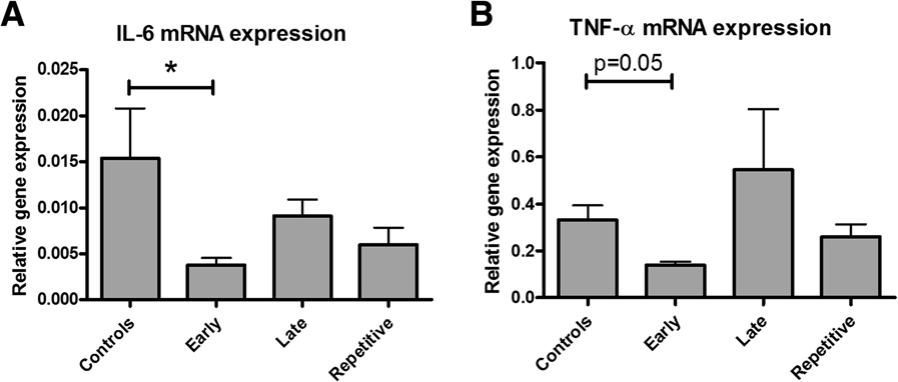
**The effect of ARA290 on inflammation.** Early ARA290 treatment reduced mRNA expression of markers of inflammation. IL-6 expression was significantly reduced by early treatment compared to controls (**A**, p < 0.05), while TNF-α expression showed a comparable tendency **(B)**. The anti-inflammatory effects of late- or repetitive treatment were less pronounced.

**Figure 3:**
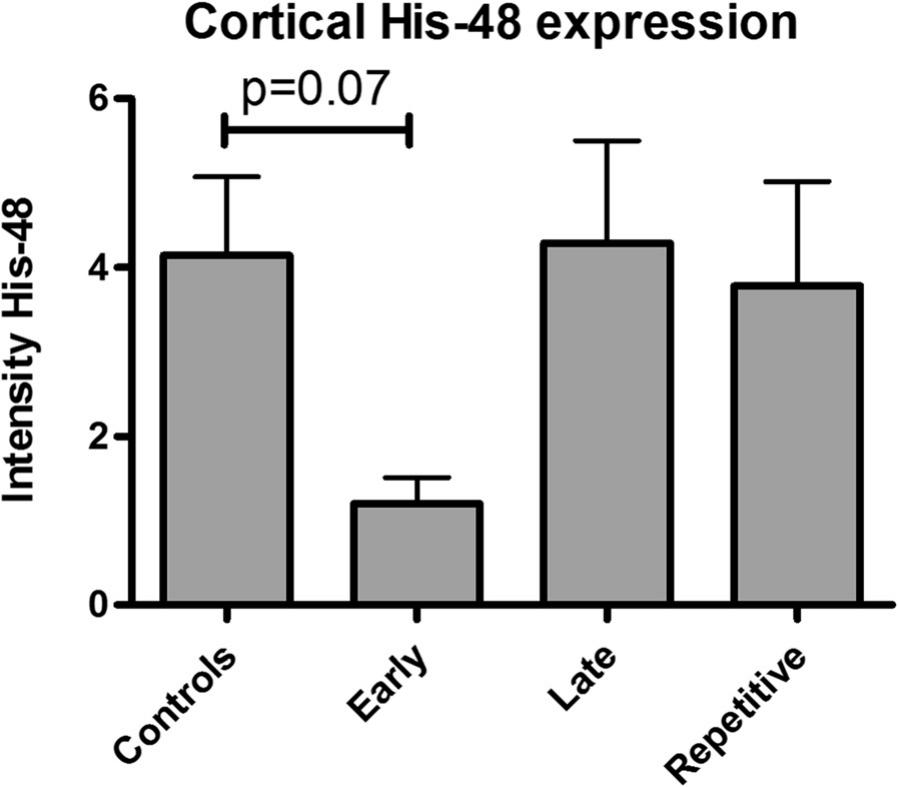
**The effect of ARA290 on granulocyte infiltration.** Early ARA290 treatment tended to reduce cortical His-48 expression as a marker of granulocyte infiltration.

qRT-PCR analyses was used to measure the expression of markers of renal inflammation at three days post-reperfusion. Early ARA290 treatment at one hour post-reperfusion significantly reduced IL-6 mRNA expression compared to the controls (Figure 2A). Renal expression of TNF-α tended to be reduced in early treated animals (Figure 2B). Late and repetitive treatment did not significantly influence mRNA expression of both inflammatory markers (Figure 2A and B). Granulocyte infiltration was evaluated by His-48 staining at three days post-reperfusion. Early ARA290 treatment tended to reduce His-48 expression in cortical tissue. Late- or repetitive treatment did not affect His-48 expression (Figure 3).

### Morphology

**Figure 4:**
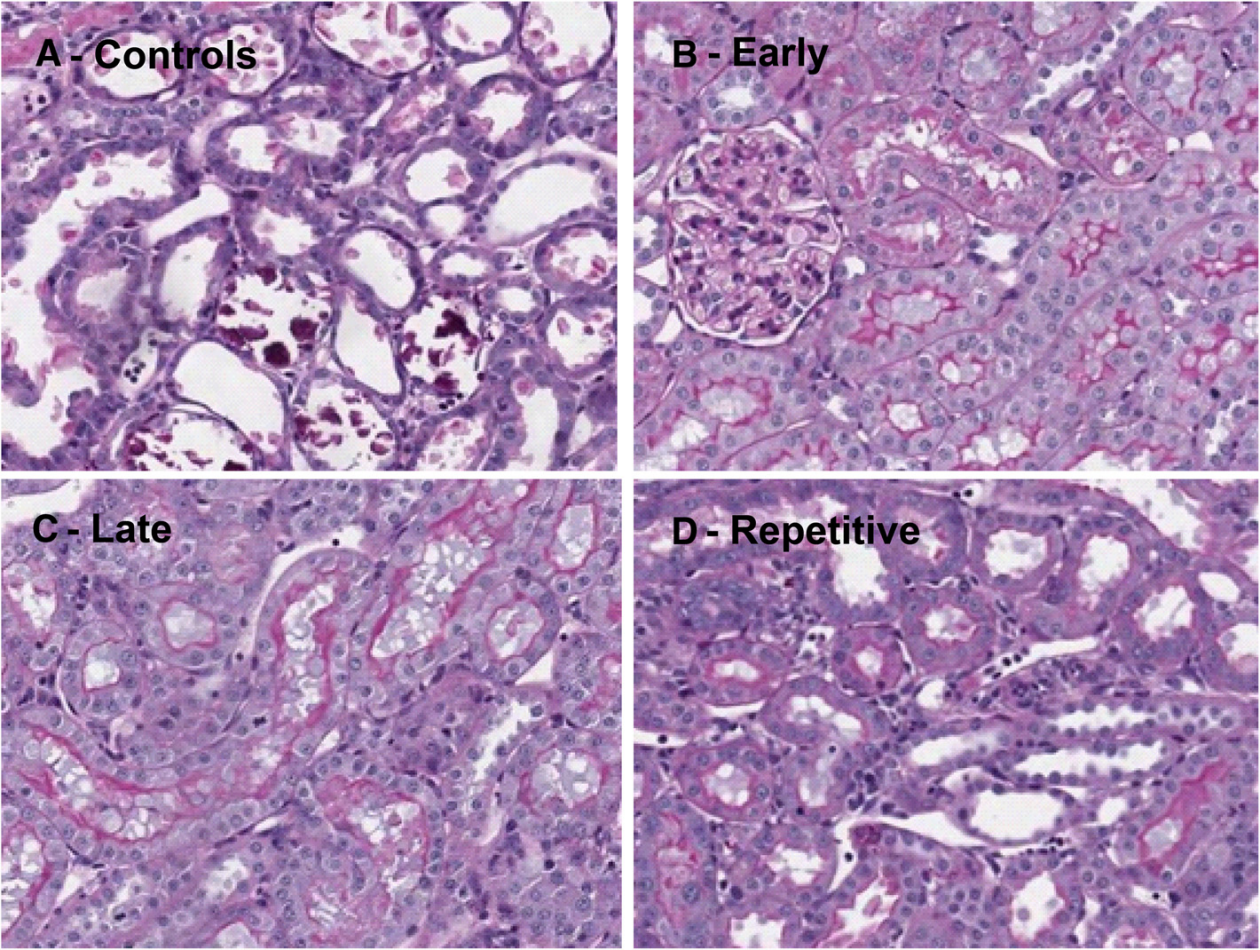
**Renal morphology by PAS staining.** ARA290 preserved renal morphology. In controls massive tubular dilatation, -necrosis and -debris was observed **(A)**. Especially, early ARA290 treatment resulted in a distinct reduction of tubular damage **(B)**. Late- **(C)** and repetitive treatment **(D)** slightly reduced renal damage.

Three days post-reperfusion animals of the control group showed severe renal damage, evidenced by tubular dilatation, cell death and cellular debris in renal tubuli (Figure 4A). Hardly any structural damage was found in the early treatment group (Figure 4B). In the groups treated at four hours (Figure 4C) or one and four hours (Figure 4D) post-reperfusion we observed slightly reduced renal damage compared to the controls.

### Acute kidney injury

**Figure 5:**
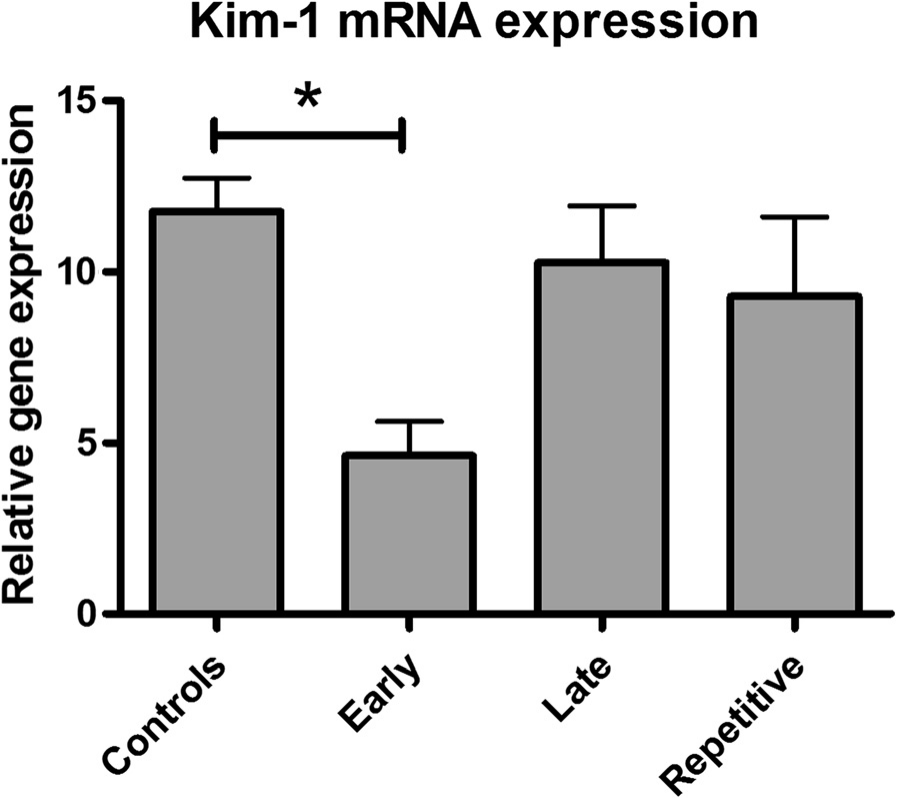
**The effect of ARA290 on Kim-1 mRNA expression.** Early ARA290 treatment reduced Kim-1 expression at three days post-reperfusion indicating reduced acute kidney injury (p < 0.05). Late- or repetitive treatment did not reduce Kim-1 mRNA expression.

**Figure 6:**
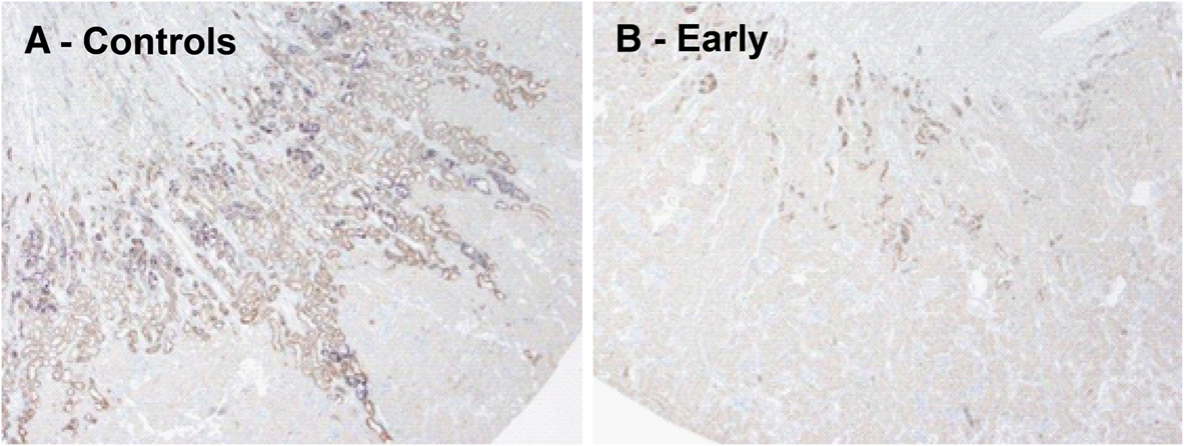
**The effect of ARA290 on Kim-1 protein expression.** In line with Kim-1 mRNA expression, early ARA290 treatment reduced tubular Kim-1 protein expression. Representative sections of control- **(A)** and early ARA290 treatment **(B)** are shown.

**Figure 7:**
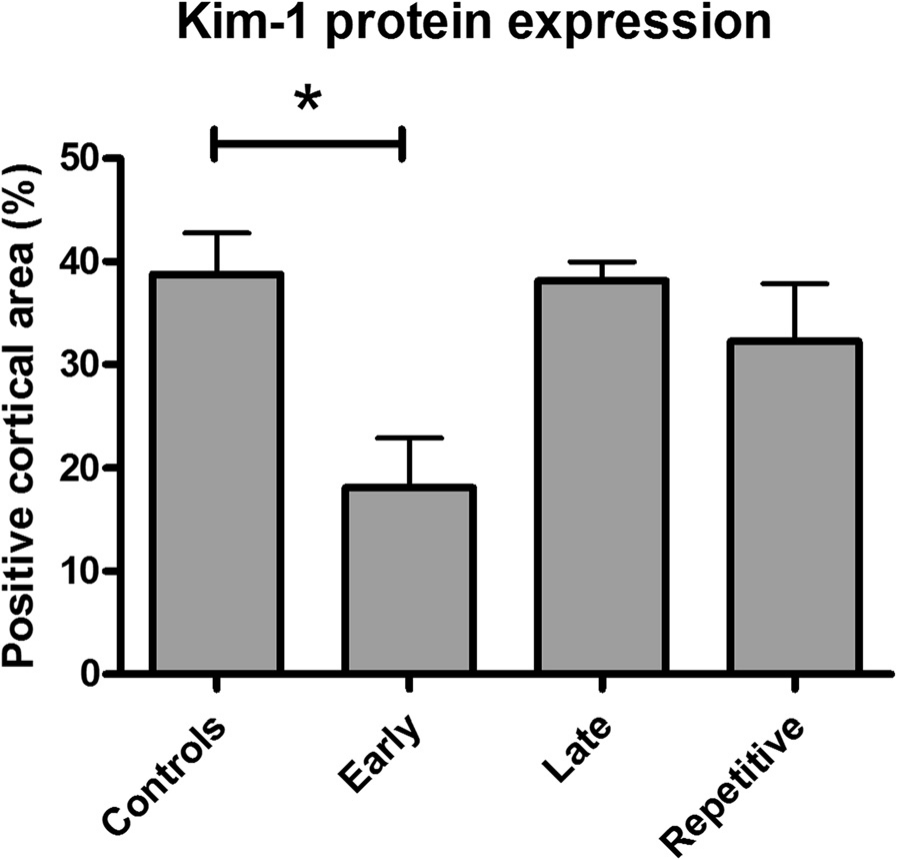
**Quantitative analysis of Kim-1 protein expression.** Early ARA290 treatment significantly reduced cortical Kim-1 protein expression (p < 0.05).

Kim-1 mRNA expression, a marker of acute renal injury, was reduced in the early treatment group compared to the controls. No significant differences between late- or repetitive treatment and the controls were found (Figure 5). Kim-1 protein expression was predominantly observed in the distal tubuli. According to Kim-1 mRNA expression, early ARA290 treatment reduced cortical Kim-1 protein expression (Figure 6A and B, Figure 7).

### Structural damage

**Figure 8:**
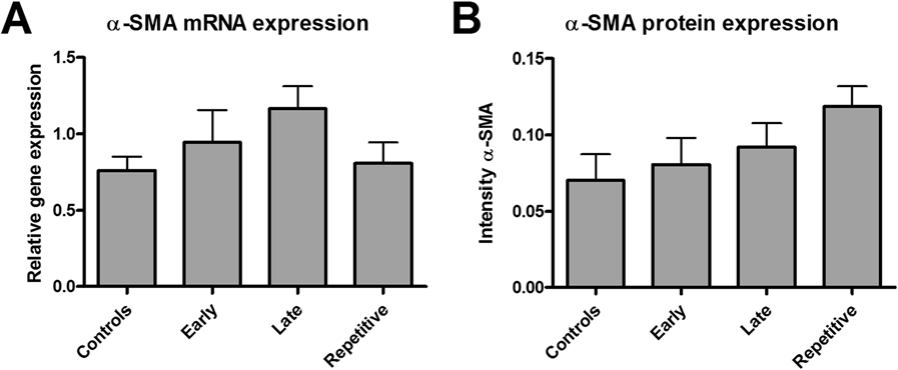
**The effect of ARA290 on structural damage.** No differences were found between controls or ARA290 treated groups in α-SMA mRNA **(A)** or cortical protein expression **(B)**.

To investigate the effect of ARA290 on structural damage we measured mRNA and protein expression of α-SMA, a marker of pre-fibrotic changes. None of the three treatment regimens influenced mRNA expression of α-SMA (Figure 8A). This result was confirmed by quantification of immunohistochemical staining of α-SMA in cortical tissue. ARA290 treatment did not reduce interstitial α-SMA expression as measured at three days post-reperfusion (Figure 8B).

## Discussion

In this study the renoprotective capacities of ARA290 were tested in a rodent model of renal I/R injury. We specifically focussed on timing and the anti-inflammatory capacities of post-reperfusional treatment of ARA290. ARA290 improved renal function, reduced inflammation and acute kidney injury three days following renal I/R. These results are in line with three recent studies showing attenuation of renal I/R injury by ARA290 [17-19]. Early administration of ARA290 after reperfusion appears to be most effective to attenuate renal I/R injury.

The anti-inflammatory effects are widely described for EPO mediated cytoprotection [5,20,21]. Patel et al. already showed that ARA290 inhibits pro-inflammatory pathway GSK-3β [18]. In our porcine renal I/R model ARA290 tended to reduce acute inflammation measured fifteen minutes after the first dose administration [19]. In this study we confirmed these anti-inflammatory effects as measured by reduced IL-6 and TNF-α mRNA expression. In line with the RT-PCR analyses early ARA290 treatment tended to reduce granulocyte infiltration. Furthermore, ARA290 reduced kim-1 expression indicating reduced acute kidney injury [22]. Based upon these results, this study confirmed the alleged anti-inflammatory effects of non-erythropoietic EPO derivative ARA290.

The direct effect on renal function, expressed by reduced serum creatinine levels, can be explained by increased eNOS phosphorylation as eNOS is known as a regulator of the renal function [9]. An eNOS knock-out model showed that eNOS is essential for attenuation of renal I/R injury by EPO [7,8]. eNOS phosphorylation is reduced in the first 6 hours following renal I/R injury and restored to normal levels at 24 hours after reperfusion [23]. This suggests that the optimal window of treatment of EPO mediated cytoprotection is in the first 6 hours. As eNOS phosphorylation levels are normalized after 24 hours, measuring eNOS phosphorylation at three days post-reperfusion is relatively late. Therefore, the effect of ARA290 on eNOS phorsphorylation has not been determined in this study. Patel et al. showed that EPO and ARA290 are able to increase eNOS phosphorylation following renal I/R [18]. In our porcine renal I/R study we showed that ARA290 increases nitrite and nitrate clearance in the first 24 hours post-reperfusion indicating increased nitric oxide synthase activity [19]. Three earlier I/R studies in which ARA290 has been tested, used different timing regimens. In mice I/R, treatment was administered at repetitively at one minute, six hours and twelve hours post-reperfusion [17]. Plasma creatinine, urea and ASAT were significantly reduced 24 hours post-reperfusion [17]. In a rat I/R model ARA290 treatment at six hours post-reperfusion reduced serum creatinine levels [18]. Repetitive treatment at zero, two, four and six hours post-reperfusion improved renal function and reduced structural damage in a porcine I/R model [19].

We therefore investigated the effect of early-, late- and repetitive administration of ARA290 in the reperfusional phase. ARA290 was administered at one hour, four hours or one and four hours post-reperfusion. Only ARA290 administered at one hour post-reperfusion induced significant renoprotection. In contrast, Patel et al. showed that ARA290 is protective in rats when administered even 6 hours post-reperfusion and in porcine renal I/R ARA290 was renoprotective when administered repetitively at 0, 2, 4 and 6 hours post-reperfusion [18,19]. The low number of animals is a limitation of this study. Especially since only four animals were included in the control group, due to technical failures. However, in all treatment groups the number of animals was similar (N = 6). This means also power to show differences between controls and treatment groups was equal. The main outcome parameter, serum creatinine, was significantly reduced by early ARA290 treatment. Observing the tendency of reduced serum creatinine levels by repetitive and late ARA290 treatment, the number of animals may have been too low to show significant differences in these groups. It is unlikely that the accumulative dose impairs renoprotective capacities of repetitive ARA290 treatment as the half-life is only several minutes. Possibly secondary activation of the EPOR_2_-βcR_2_ receptor complex is detrimental in rats. However, porcine models are more suitable to further investigate timing of ARA290 for translation to the human setting as pigs are physiologically more comparable to human. This study shows the importance of timing and gives direction to design of large animal models testing the protective capacities of ARA290 against renal I/R injury.

In this study the animals were euthanized three days post-reperfusion. No differences were found in α-SMA expression in contrast to the porcine I/R model [19]. Morphologically, ARA290 treatment reduced tubular dilatation and cell death indicating reduced structural damage. Villanueva et al. showed that α-SMA expression can already be up-regulated 24 hours after renal ischemia/reperfusion [24]. Seven days post-reperfusion ARA290 prevented increased expression of α-SMA following porcine renal I/R [19]. Although α-SMA expression can be up-regulated early following I/R, three days post-reperfusion might have been too early to measure changes in this study.

## Conclusions

In conclusion, this study shows the renoprotective properties of early post-reperfusion administrated ARA290. Early administration at one hour post-reperfusion is distinctly more effective than treatment at four hours post-reperfusion. Besides, this study shows the anti-inflammatory capacities of ARA290. Considering the renoprotective and anti-inflammatory effects of ARA290, it is a promising treatment to attenuate renal I/R injury following renal transplantation. Especially, DCD- or older DBD kidneys at risk for DGF and PNF are potential targets of ARA290 mediated cytoprotection. These results warrant further investigation of the protective effects and timing of ARA290 in large animal models of renal transplantation.

## Abbreviations

α-SMA: α-smooth muscle actin; βcR: β common receptor; DBD: Donation after brain death; DCD: Donation after cardiac death; DGF: Delayed graft function; eNOS: Endothelial nitric oxide synthase; EPO: Erythropoietin; EPOR: Erythropoietin receptor; GFR: Glomerular filtration rate; GSK-3β: Glycogen synthase kinase-3β; IL-6: Interleukin-6; I/R: Ischemia/reperfusion; JAK-2: Janus kinase-2; Kim-1: Kidney injury molecule-1; pHBSP: Pyroglutamate helix B-surface peptide; PNF: Primary non function; qRT-PCR: Real-time reverse transcription polymerase chain reaction; TNF-α: Tumour necrosis factor-α.

## Competing interests

All authors declare no conflict of interests. ARAIM pharmaceuticals (Ossining, USA) provided an unrestricted grant for this study.

## Authors’ contributions

WGR: Design of the study. Analysis and interpretation. Drafting of the article. GJN: Interpretation of data. Revised the article. HG: Design of the study. Analysis and interpretation. Revised the article. PJO: Design and performance of the study. RJP: Design of the study. Revised the article. HGDL: Design of the study. Analysis and interpretation data. Revised the article. All authors read and approved the final manuscript.
